# Supplementary material for: The Dual Prey-Inactivation Strategy of Spiders—In-Depth Venomic Analysis of Cupiennius salei
Source: Toxins (Basel). 2019 Mar 19;11(3):167. doi: 10.3390/toxins11030167 (PMC6468893; doi:10.3390/toxins11030167)
Supplement: Supplementary file 1 [file toxins-11-00167-s001.zip › Supplementary Fig S1.1-S1.19/Supplementary Fig S1.6.pdf]

Supplementary Fig S1.6

CLUSTAL O(1.2.4) multiple sequence alignment: Peptidylglycine α-amidating monooxygenases

```
PAM_CUPSA                  MKSESTFNFIAKIWVLGFLTAAC--------FADGETSKK-SLLMPDVQPKVPETYLCTA
PAM_ALOMA                  MKSESNFKFIAKCLVVGLLTTIC--------VVEGETTKKLPLLMPDVQPKVPETYLCTA
PAM_VIRFA                  -------NFIVTALSIGYLAIDLFI--AVSFTKAEESSRKLPLLMPDVQPKVPETYLCTA
gi_XP_015915160.1_PARTE    ------MKLLSVIYAVKIIFSILYLTTARQVKPNDDGRKYLPLLMPDVQPVVKETYLCTA
tr_JAT91064.1_TITOB        ---------MAKH----LFVSSLFLI---QLSSLCFAYRKLEMLMPNVQPLHNETYFCTS
gi_XP_013784032.1_LIMPO    ------MELL-SIWLV-------FIL----TITHSLATVKFPMHMPDVQPTQKETYLCTA
                                    :                                : **:***   ***:**:

PAM_CUPSA                  FTMPKSDFEYVVAFTPNASMHTAHHILIYGCEIPGQWERDSPRLVWDCGEMSGQQSGYLR
PAM_ALOMA                  FTMPKSEFEYVVAFTPNASMHTAHHILIYGCEIPGQWERDSPRLVWDCGEMSGQQSGYLR
PAM_VIRFA                  FTMPKYDFEYVVGFTPNASMHTAHHILIYGCDIPGQWQRDSPRLVWDCGEMSGHQSGYLR
gi_XP_015915160.1_PARTE    FRMPQTDFKYIVEFTPNASMHVAHHILIYGCEIPGRWERDTPRLVWECGEMSGSKNSYIQ
tr_JAT91064.1_TITOB        FKMNKHEHEYVVKFEPNATMHVAHHILIYGCEEPGLVQRDTPRIVWNCGEMMGSNSEYVS
gi_XP_013784032.1_LIMPO    FKMPAREHQYIVEFEPNATMHTAHHILIYGCTTPGFWAMDDPRAVWDCGEMAGGKSEYRR
                           * *   :.:*:* * ***:**.*********  **    * ** **:**** * :. *

PAM_CUPSA                  GPTCSSGSQIIYAWAKDAPPLKLPEGVAFKVG-KDTGINYLVLQVHYAHVDKFLGGETDN
PAM_ALOMA                  GPTCSAGSQIIYAWAKDAPPLKLPEGVGFKVG-RNTGINYLVLQVHYAHVDKFLSGETDN
PAM_VIRFA                  GPTCSAKSQIIYAWAKDAPPLKLPEGVGFRVG-RDTGINYLVLQVHYAHVDKFLGGETDN
gi_XP_015915160.1_PARTE    GPTCNTGSQIIYAWAKDAPPLSLPDGVAFKVG-RNTGINFLVLQVHYAHVEKFIMGGKDN
tr_JAT91064.1_TITOB        APICAEGSQVIYAWARDAPALNLPEGVGFKVG-KGSGINYLVLQVHYADVTRFLNGGTDH
gi_XP_013784032.1_LIMPO    APTCASGSQVIYAWARDAPKLTLPEGVGFKVGGPDTGIEYLVLQVHYANVDKFLNGATDD
                           .* *   **:*****:*** *.**:**.*:**  .:**::********.* :*: * .*.

PAM_CUPSA                  SGIILSLLPSSTDKVTKTAGVYLLGTNGMIRAREEEHFEVACRINDPVVLHPFAFRTHTH
PAM_ALOMA                  SGIILSVLPSSTKKVTKTAGVYLLGTNGMIRAREEEHFEVACRINDPITIHPFAFRTHTH
PAM_VIRFA                  SGIVLSLLPSTSKKVTKSAGVYLLGTNGMIRAREEEHFEVACRINEDVVLHPFAFRTHTH
gi_XP_015915160.1_PARTE    SGIILTMLPSTTDKVTKAAGVYVLGTGGEIRAGEEEHFETACKINEPLKLHPFAFRTHTH
tr_JAT91064.1_TITOB        SGIILTLLPGSDKSVTKRAGVYLLGTSGFIRAKQLEHMETACKIDKRLVIHPFAFRTHTH
gi_XP_013784032.1_LIMPO    SGVILSTLPGTTNKVNKRAAILLLGTGGVIPAHREEHMETACTIQEPVVLHPFAFRTHTH
                           **::*: **.: ..*.* *.: :***.* * * . **:*.** *:. : :**********

PAM_CUPSA                  ALGKVVSGYKIDKD-GKWHMIGKHNPQEPQMFYPIEDKDLTIEYGDIVAARCTMYNFRSR
PAM_ALOMA                  ALGKVVSGYKIDKD-GKWQLIGKHNPQEPQMFYPIEDKHLTIEYGDIVAARCTMYNFRNR
PAM_VIRFA                  VLGKVVSGYKIDKD-GKWQQIGKHNPQEPQMFYPVENKDMTVEYGDIVAARCTMYNFRQK
gi_XP_015915160.1_PARTE    ALGKVVSGYKIDKE-GKWHFIGKHDPQEPQMFYPVEDKSLTIEYGDVVAARCTMYNFRDR
tr_JAT91064.1_TITOB        ALGKAVSGYVIDRDSGKWKLIGKHNPLEPQMFYPVKDKHLVIQEGDVLAARCTMYNFRNR
gi_XP_013784032.1_LIMPO    QLGKAVAGYVMRKN-GMWERIGKHDPLQPQMFYPVNK-KISIFQGDVVAARCTMKNFRDV
                            ***.*:** : :: * *. ****:* :******::.  : :  **::****** ***.

PAM_CUPSA                  DTYIGPTGDDEMCNFYMMYYIDGDKPMSEKYCFSNGPNNYYWDRDSNVGYVPQEVDEGAS
PAM_ALOMA                  DTYIGPTGDDEMCNFYMMYYVEGDKPMSEKYCFSNGPNNYYWDRDSNIGYVPPEVDEGAS
PAM_VIRFA                  DTYVGPTAEDEMCNFYMMYYVEGDKPMSDKYCFSNGPNSYYWDRDILLGYVPQEVDEGAS
gi_XP_015915160.1_PARTE    DTYIGSTGEDEMCNFYMMYYVDGDKPMEEKYCFTSGPPVYYWSRDFKVGSVPKNIDLSAS
tr_JAT91064.1_TITOB        ITSIGSTAEDEMCNFYMMYYVEGDEILDDKYCFSMGPPAYYWDNDELVGDVPKWVDEDAS
gi_XP_013784032.1_LIMPO    TTRVGLTGNDEMCNFYIMYYVDGDRILTNKYCFTAGPPGYRWRYDPSIGDVPPDVDADAS
                            * :* *.:*******:***::**. : :****: **  * *  *  :* **  :* .**

PAM_CUPSA                    DLEN-
PAM_ALOMA                    DLEN-
PAM_VIRFA                    LLDN-
gi_XP_015915160.1_PARTE      TI---
tr_JAT91064.1_TITOB          SLRE-
gi_XP_013784032.1_LIMPO      SLDDL
                              :
```

Percent Identity Matrix - created by Clustal2.1

```
     1: PAM_CUPSA                 100.00   83.82   90.93   72.75   64.12   61.24
```

2: PAM_ALOMA	90.93	83.29	100.00	72.54	65.98	61.06
3: PAM_VIRFA	83.82	100.00	83.29	68.09	62.90	60.41
4: gi_XP_015915160.1_PARTE	72.75	68.09	72.54	100.00	62.50	61.00
5: tr_JAT91064.1_TITOB	64.12	62.90	65.98	62.50	100.00	62.61
6: gi_XP_013784032.1_LIMPO	61.24	60.41	61.06	61.00	62.61	100.00

Proteomic coverage trypsin

>PAM_CUPSA peptidylglycine alpha-amidating monooxygenase

MKSESTFNFI AKIWVLGFLT AACFADGETS K**KSLLMPDVQ PKVPETYLCT AFTMPK**SDFE YVVAFTPNAS MHTAH**HILIY**
GCEIPGQWER DSPR**LVWDCG EMSGQQSGYL RGPTCSSGSQ IIYAWAKDAP PLKLPEGVAF KVGKDTGINY LVLQVHYAHV**
DKFLGGETDN SGIILSLLPS STDKVTKTAG VYLLGTNGMI RAREEEHFEV ACRINDPVVL HPFAFRTHTH ALGK**VVSGYK**
IDKDGKWHMI GKHNPQEPQM FYPIEDKDLT IEYGDIVAAR CTMYNFRSRD TYIGPTGDDE MCNFYMMYYI DGDKPMSEKY
CFSNGPNNYY WDRDSNVGYV PQEVDEGASD LEN

Proteomic coverage chymotrypsin

>PAM_CUPSA peptidylglycine alpha-amidating monooxygenase

MKSESTFNFI AKIWVLGFLT AACFA**DGETS KKSLLMPDVQ PKVPETY**LCT AF**TMPKSDFE YVVAF**TPNAS
MHTAHHILIY **GCEIPGQWER DSPRLVWDCG EMSGQQSGYL RGPTCSSGSQ IIYAWAKDAP PLKLPEGVAF KVGKDTGINY**
LVLQVHYAHV DKFLGGETDN SGIILSLLPS STDKVTKTAG VYLLGTNGM**I RAREEEHFEV ACRINDPVVL HPFAFRTHTH**
ALGKVVSGYK IDKDGKWHMI GKHNPQEPQM FYPIEDKDLT IEYGDIVAAR CTMYNFRSRD TYIGPTGDDE MCNFYMMYYI
DGDKPMSEKY CFSNGPNNYY WDRDSNVGY**V PQEVDEGASD LEN**

Supplementary Fig S1.6 Multiple sequence alignment of PAM from the venom glands of *C. salei* (PAM_CUPSA [MH766628]), *A. marikovskyi* (PAM_ALOMA), *V. fasciatus* (PAM_VIRFA), *P. tepidariorum* (gi_XP_015915160.1_PARTE), the scorpion *Tityus obscurus* (tr_JAT91064.1_TITOB), and the horseshoe crab *L. polyphemus* (gi_XP_013784032.1_LIMPO). Signal peptides are marked in black characters except for *P. tepidariorum*, where no cutting site could be determined with SignalP (highlighted in gray). Signal peptides are in black characters, cysteines are colored in white with a black box, positively charged amino acids in pink, negatively charged amino acids in blue, hydrophobic amino acids in red and polar amino acids in green. The copper type II, ascorbate-dependent monooxygenases signature 2 is highlighted in yellow.
